# The Relationship between Physical Activity and Academic Achievement in Multimodal Environment Using Computational Analysis

**DOI:** 10.1155/2022/9418004

**Published:** 2022-08-30

**Authors:** Lingshu Li, Li Zhang

**Affiliations:** ^1^Department of Physical Education, Shanghai International Studies University, Shanghai 201620, China; ^2^Department of Physical Education, China Pharmaceutical University, Nanjing, Jiangsu 211112, China

## Abstract

Health has always been recognized as the imperative parameter to excel in any field whether professional or personal. People with sound health having regular habit of physical activities show more potential in their professional and personal lives than the people who do not participate in the physical activities. Many state-of-the-art studies exist in the literature where researchers have proved the significance of physical activities as supportive treatment for the existing ailments and in improving the overall health of human beings. Our research aims at accessing the correlation between physical activities carried out by the students and its impact on the success rate of academic achievements. The study is using computational techniques to investigate the relevance of physical activities on the academic achievements of middle school children. The study employs data mining techniques for processing the data. The computational methods are used in a multimodal environment where the surrounding parameters of the environment are considered before performing computational techniques on the subjects (participants in terms of sample for the study). In this cross-sectional study, we have considered the data on various physical activities such as aerobic fitness, running, playing, and participation in extracurricular activities. After collection of data in a real multimodal environment from middle school students, the data preprocessing is performed to handle the missing values. Then, the computational techniques are applied in a step-by-step approach using regression and the bootstrap methods to examine the data and predict the outcome. The correlation is assessed between academic achievements and physical activities. The outcome predicts that physical activities promote the success rate of academic achievements including extra-curricular activities.

## 1. Introduction

### 1.1. Background Study

As the focus on academic achievement has increased in the schools, physical activities have reduced. Health is the most important criterion, which impacts the human performance in all fields. Diet and physical activities play equally important role in the overall health of human beings [1]. Childhood obesity is a dominant threat to public health in the United States, which has been linked with physical health problems [2] and poorer academic achievement (AA) [3]. Children's health and AA can be improved through physical activity (PA) and aerobic fitness. To date, however, there is limited understanding of how indicators (PA, aerobic fitness, and attention) operate together jointly and separately to influence AA. Some inconsistencies in researches on the relationship between PA, aerobic fitness, and AA, which could be attributed to measure and methodological issues (e.g., measuring only PA or aerobic fitness and inadequate control of socio-economic variables); findings were either a positive or a negative association. The latter is rarely reported [4–6]. Therefore, the purpose of this study was to investigate the relation between PA and AA among middle school students.

### 1.2. Existing Literature

PA exerts the greatest effect on middle school and elementary students [7]. Among this population, active children generally show healthier cardiovascular features, aerobic fitness, and PA [8]. The effect of PA on cognitive ability was task dependent and was most evident in perceptual skills, followed by IQ [9, 10]. Previous studies have shown that PA has effects on enhancing angiogenesis, increasing oxygen saturation and glucose delivery, improving cerebral blood flow, and increasing neurotransmitter levels, which is conducive to children's cognitive function [11]. A study of 248 children (aged 8 to 11 years) indicated that PA was associated with VO2 peak, a measure of aerobic fitness, in both males and females [12]. However, there does not exist any study that correlate the relationship between PA, aerobic fitness, and AA. In separate models, the associations between PA and AA have been evaluated. PA, increasing physical effort, and social engagement can challenge executive functions and academic achievement. However, it fails to statistically make clear possible collinearity [13]. Some researchers have found that there is a lack of a relationship or a negative relationship between PA and AA. Moreover, the association between PA and aerobic fitness indicates a mediating effect of AA [14]. In reference [15], authors state that the influence of PA on AA may extend through the impact of PA on aerobic fitness. Aerobic fitness is an indicator of physical performance. Some studies have illustrated that aerobic fitness can not only promote hippocampal cerebral blood flow but also contribute to brain function and structure development [16]. How these measures (PA, aerobic fitness, and cognitive ability) interact and consequently influence AA is not yet clearly understood. Attention, as a major part of cognitive ability, has never been explored in the relationship between PA and AA. Without higher levels of attention and aerobic fitness, achieving and sustaining at least a moderate PA level might be precluded. Furthermore, there has been some evidence to prove that compared to physical activity, these physiological features are associated with academic achievement and cognitive function [17–20].

In reference [21], authors have carried out a survey to summarize the meta-analyses about the effect of PA on AA of school-going kids and teenagers. As per their findings, PA had shown null or small-to-medium positive effects on AA. Regular PA showed a medium-positive effect on AA, and rigorous PA did not reveal any benefits. In reference [22], authors conclude through their study that the PA and physical fitness are positively correlated with the AA of teenagers. They also conclude that the physical fitness criteria are closely related to the AA was cardio-respiratory fitness. In reference [23], authors identify two large gaps in the research carried out to correlate PA and AA: identifying the level and type of PA needed. The optimal type of PA to improve academic outcomes is also unknown. In reference [24] authors revealed that the overall effect of PA on AA was tilting towards positive; however, it was smaller than the total indirect impact through mediators. An indirect impact is the aggregate of the impact of self-confidence and depression, but the self-esteem factor was found to be the strongest mediator between PA and AA. Therefore, the present study aimed to evaluate the relationship between PA and AA and included attention, aerobic fitness, and other possible factors as mediators.

### 1.3. Major Highlights of the Proposed Study

1. The cross-sectional study uses computational techniques to investigate the relevance of PA on the AA of middle school children and employs data mining techniques for collection of data and processing of data.
2. The study is aimed to identify the effect of PA on the academic performance of the school going kids.
3. The study also evaluates the correlation between MVPA (moderate-to-vigorous PA) and performance for mathematics scores of the participants
4. The study uses multiple regression (MR) analysis and bootstrap analysis (BA) to conduct the experimental analysis.

The next section discusses about the proposed method.

## 2. Proposed Method

The proposed study uses regression analysis and BA to conduct the experimental analysis. The samples of the school going kids are collected to prepare the dataset. In order to evaluate the data, methods such as MRA and BA are applied as explained further in this section.

### 2.1. Multiple Regression Analysis (MRA)

It is an assessment of MR in statistics. It is an advancement of linear regression (LR). In statistics, LR is the used to forecast the value of one variable dependent on another variable. In MR, dependent variable depends on values of two or more variables. The MR analysis (MRA) estimates the information using regression.

### 2.2. Bootstrap Analysis (BA)

The bootstrap method is a resampling technique. It is used for the estimating the statistics on a population by sampling a dataset with replacement. It can be used to estimate mean or standard deviation (summary statistics).

This study uses MR analysis and BA to conduct the experiment of 176 seventh and eighth-grade students from USA and analyze the relationship between their mathematics scores and other measurements stated below. The following part will specifically introduce the research participants, measurement, study procedure, and data analysis process.

### 2.3. Participants or Subjects

About 176 middle school students (from the 7th and 8th grade) from the school located in Salt Lake City, UT, USA are selected as subjects according to the cluster sampling technique [17–19]. Among them, 93 students were boys and 93 students were girls. An analytical cross-sectional analysis was performed. The study procedure was permitted by the institutional review board and the district research board. Written informed consent was taken from parents and students to conduct this research study.

### 2.4. Measurement

The specification of the measurement for each of our parameters has been explained as follows:

#### 2.4.1. Physical Activity (PA)

PA was estimated (maximum of 7 days per student) with a pedometer (NL-1000). Two PA measures were used as an independent variable: average number of steps for 1 week (steps) and average duration of moderate-to-vigorous PA (MVPA) for 1 week [16].

#### 2.4.2. Body Mass Index (BMI)

Height and weight were measured using a portable scale with an accuracy of 0.1 kg (BD-590; Tokyo, Japan) and a portable stadiometer with an accuracy of 0.01 m (Seca 213; Hanover, MD, USA), respectively. BMI was calculated (kg/m^2^) and standardized; BMI z-score was obtained.

#### 2.4.3. Aerobic Fitness

Using the PACER program, participants were asked to run as long as possible at a specified pace. They had to run back and forth across a 20 meter space at a fast pace every minute, with one point being added for every 20 meters [17].

#### 2.4.4. Selective Attention

Selective attention was measured using the Stroop test. The Stroop test in the psychology is a measure of delay in reaction time. It indicates the flexibility of cognitive thinking The Stroop test was intended to measure selective attention and cognitive flexibility [18]. Our study followed the guideline according to previous studies: two types of paper cards were used to measure selective attention, and each card contained stimuli against a white background. On card A, every color's name was printed in black ink. On card B, serving as an interference card, color names did not match with ink color (e.g., black word printed in purple, green, or orange). Participants were asked to read out the words on card A as quickly as they can. For card B, they had to indicate the color of the printed word, not the word itself. The time taken to complete the task (all errors were corrected promptly without stopping the stopwatch) represented selective attention 1 (card A) and selective attention 2 (card B).

#### 2.4.5. Sustained Attention and Alternating Attention

Sustained attention and alternating attention were measured through the trail making test (TMT). The original test was developed by Partington [19]. The task involves connecting 25 consecutive targets on a piece of paper or a computer screen in a way that resembles a “connect-the-dots” puzzle played by children. The test has two parts: the first part (TMTA) is the numbers test, where participants are asked to identify the numbers sequentially; the second part (TMTB) is a test, in which the child alternates between numbers and letters (1, A, 2, B, etc.) [22]. TMTA and TMTB measure sustained attention and alternating attention, respectively. Each participant practiced before the test and the test duration was determined. The examiner corrected errors immediately, without stopping the chronometer. The shorter the duration, the better is the sustained attention and alternating attention.

#### 2.4.6. Academic Achievement (AA)

AA was measured based on the students' mathematics score. The New York Test, which was designed by the New York State Education Department for testing students' mathematics level, was employed. There were 30 questions in the grade 7 edition and 27 in the grade 8 edition.

### 2.5. Procedure

Information about the study was shared with the students during their physical education (PE) classes. Consent forms were sent to the parents through the students; the parents had the chance to ask questions before starting the study. Child assent was completed during PE classes. During the first week, participants' height, weight, and aerobic fitness through a running test (PACER) were determined. During the second week, the PA test (i.e., steps and MVPA) was performed; measurement was obtained for 7 consecutive days. Starting from Monday, students started wearing a pedometer once they arrived at school and were required to wear the monitor all day until bedtime. The attention of the students was also evaluated. The participants were asked to complete the Stroop test and TMT; their mathematics scores were identified during their PE classes. BMI was calculated using height and weight measurements.

### 2.6. Data Analysis

All data were analyzed by SPSS 26.0 (IBM Corporation, Armonk, NY, USA). Variance analysis, least significant difference *t*-test, multivariate regression, and BA were conducted. These methods could help us understand the degree of the assumed positive correlation between PA and AA. We conducted the experiments with a sample size of 176 students, which was the average number in a typical public middle school in Utah; our study thus has sufficient statistical power. The sample size was also determined based on the number of schools willing to participate and their respective student membership size.

MR analysis was used to analyze the relationship between mathematics scores of grade 7 and 8 students and MVPA, number of steps, aerobic fitness, selective attention 1, selective attention 2, sustained attention, and alternating attention; the final regression equation was obtained. Regression analysis was performed by progressive stepwise regression and expressed as 95% confidence interval.

BA was used for analyzing mediating effects and was considered appropriate for the expected non-normality of the sampling distribution of the mediation effect [13]. Based on the direct and gradual effects between independent, dependent, and mediating variables, the mediating relationship was calculated for each variable.

## 3. Result Outcome

### 3.1. Descriptive Statistics of Grade 7 and 8 Students

Descriptive statistics are presented in Table 1. About 176 secondary school students enrolled in grades 7 and 8, 52.8% were female. Moreover, 52.8% were grade 7 students (48 boys and 45 girls) and 47.2% were grade 8 students (45 boys and 38 girls).

According to the World Health Organization (WHO) Growth Reference [17], pupils' BMI are divided into five categories: severely thin, thin, normal, overweight, and obese. The BMI classification of children aged 13 and 14 years was identified monthly; categories according to the WHO Growth Reference were used as standards (Table 2). The descriptive statistics of the BMI of grade 7 and 8 students are listed in Table 3.

The sample characteristics for middle school students in grades 7 and 8 are listed in Table 1.

The sample characteristics for middle school students in grades 7 and 8 are listed in Table 2.

The distribution of BMI in male and female students in grades 7 and 8 is listed in Table 3.

### 3.2. Multiple Linear Regression Analysis of PA and Potential Mediating Variables and Mathematics Scores

Using experimental research, we have measured the indicators of each student, which are forming a categorical variable of BMI and multiple continuous variables. The forward stepwise regression method was used for the dependent variable Y: math and the independent variables *X*1: MVPA, *X*2: steps, *X*3: aerobic fitness, *X*4: selective attention 1, *X*5: selective attention 2, *X*6: sustained attention, *X*7: alternating attention, and *X*8: BMI. For BMI, dummy variables were recorded: *X*8: severe thinness, *X*8: thinness, *X*8: normal, *X*8: overweight, and *X*8: obesity. Regression analysis was performed to construct a MR model to test the interconnection between PA, potential mediating variables, and total academic scores.

MVPA and steps played a common role among the seventh and eighth-grade students and always had a positive effect on mathematics scores. In males, sustained attention had an effect on mathematics scores; selective attention 1, selective attention 2, alternating attention, and BMI were eliminated in the equation. The final equation of the multiple LR of the independent and dependent variables of males is written as follows:

(1) $$\begin{array}{c} \hat{Y}=5.839+1.302X1+0.004X2+0.657X6. \end{array}$$

It could explain 47.7% of the total variation of the dependent variable (Table 4). Thus, MVPA steps and sustained attention could predict mathematics score to a certain extent. MVPA steps and sustained attention had positive effects on the improvement of mathematics scores in males. In females, the final equation of the multiple LR is expressed as follows:

(2) $$\begin{array}{c} \hat{Y}=6.327+1.610X1+0.005X2. \end{array}$$

This could explain 60.1% of the total variation of the dependent variable (Table 5). Therefore, MVPA and steps could predict mathematics score to a certain extent, and MVPA and steps had positive effects on the improvement of mathematics scores in females.

The regression coefficient of the final model of boys' regression equation is listed in Table 4.

The regression coefficient of the final model of girls' regression equation is listed in Table 5.

### 3.3. Standardized Residual Analysis of the Regression Model

**Figure 1:**
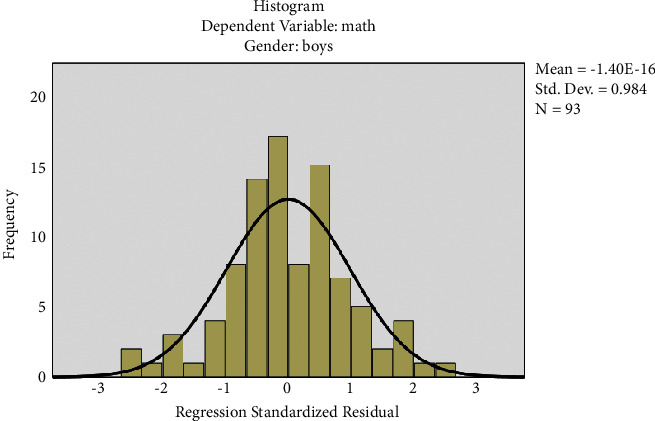
Standardized residual histogram for the regression equation of boys. The regression-standardized residuals show normal distribution, which means that the data meet the requirements of the regression equation (same is applicable for Figures 2to 6).

**Figure 2:**
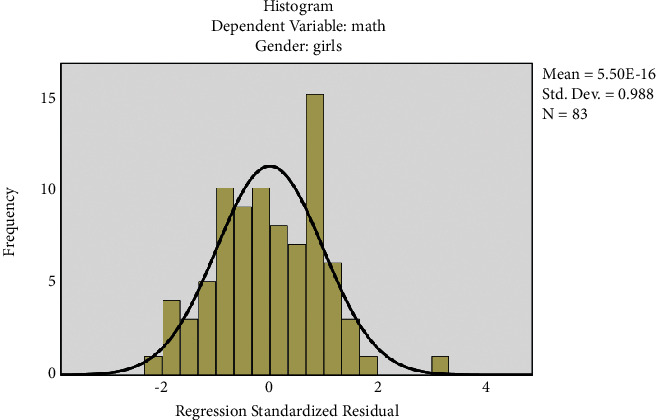
Standardized residual histogram for the regression equation of girls. The regression-standardized residuals show normal distribution.

**Figure 3:**
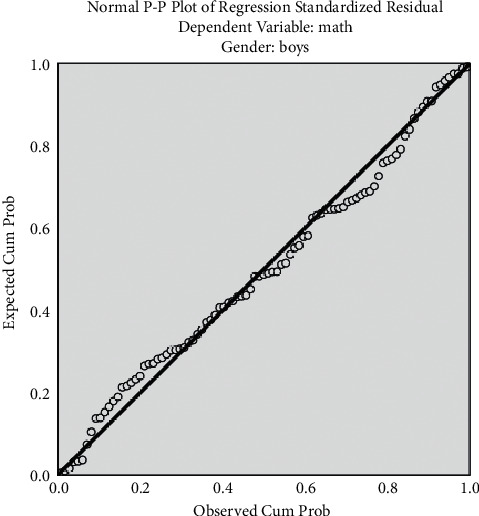
Regression-normalized residual P-P diagram of boys. The scattered point distribution of standardized residuals is close to the straight line.

**Figure 4:**
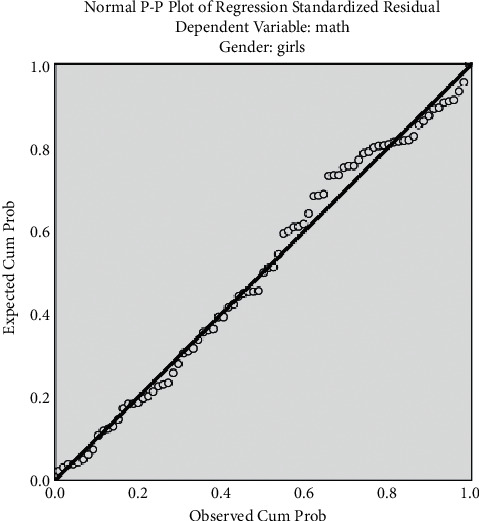
Regression-normalized residual P-P diagram of girls. The scattered point distribution of standardized residuals is close to the straight line.

The histogram has been plotted for the standardized residuals of the regression equations. The histograms of standardized residuals of the regression equations, from which standardized residuals showed normal distribution, conform to the hypothesis test condition (Figures 1 and 2). From the normal P-P graph of normalized residuals, the scattered point distribution of standardized residuals was close to the straight line, indicating that normalized residuals conform to normal distribution and residuals satisfy the conditions of multivariate LR (Figures 3 and 4).

**Figure 5:**
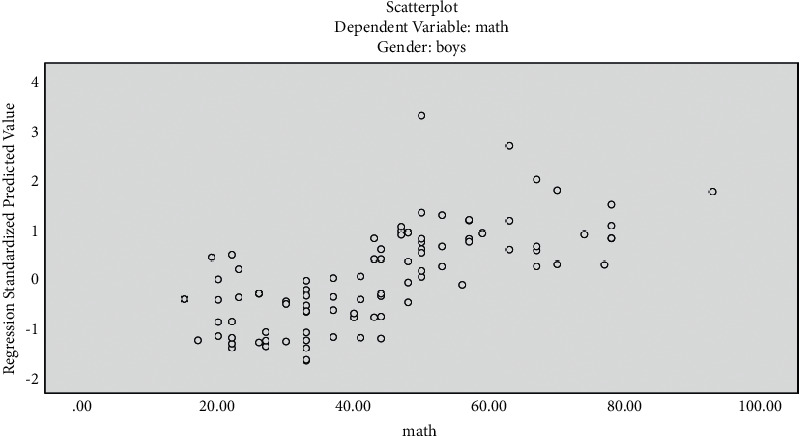
Scatter plot of regression-standardized prediction value (boys). Dependent variable is placed on the *x* axis and ^∗^ ZPRED is the *y*-axis variable. The two variables are in a straight-line trend.

**Figure 6:**
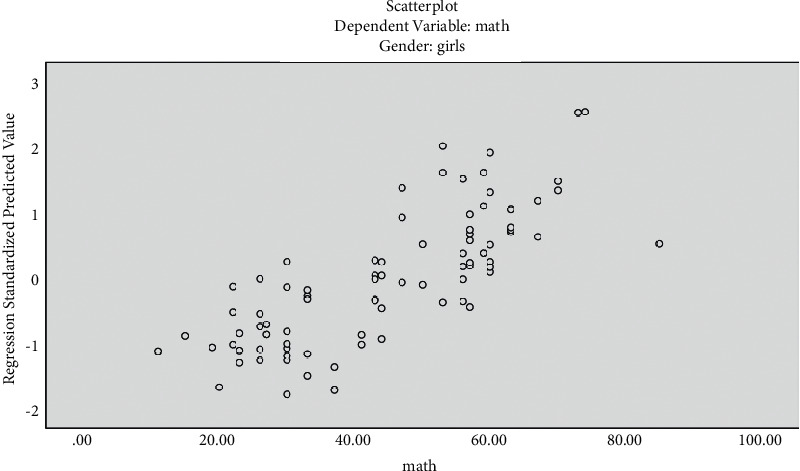
Scatter plot of the regression-standardized prediction value (girls). Dependent variable is placed on the *x* axis and ^∗^ ZPRED is the *y*-axis variable. The two variables are in a straight-line trend.

Figures 5 and 6 show scatter plots of the mathematical scores and the standardized prediction value of regression, with the dependent variable on the *x* axis and *∗*ZPRED on the *y* axis. The two variables were in a straight-line trend.

Therefore, step, MVPA, and sustained attention could predict mathematics score to a certain level. In males, the lower the average number of steps, the shorter the duration of MVPA, and the shorter the sustained attention time, the worse the mathematics score, which is contrary to the assumption. Moreover, the greater the average numbers of steps and longer the duration of MVPA, the better the total mathematics score. In females, sustained attention time did not influence the mathematics score. However, similar to males, the greater the average numbers of steps and longer the duration of MVPA, the better the total mathematics score.

Figure 1 shows the standardized residual histogram for the regression equation of boys.

Figure 2 shows the standardized residual histogram for the regression equation of boys.

Figure 3 shows regression-normalized residual P-P diagram of boys.

Figure 4 shows regression-normalized residual P-P diagram of girls.

We plotted the scatter plot of regression-standardized prediction values for boys and girls as shown in Figure 5. Figure 5 depicts scattered plot of regression-standardized prediction value (boys).

Figure 6 shows the scatter plot of the regression-standardized prediction value (girls).

### 3.4. Test for Mediating Effect

We have carried out the BA to find out the mediating effect of MVPA on the mathematical achievement of students and the same has been explained as below.

#### 3.4.1. Analysis of the Mediating Effect of MVPA on the Mathematical Achievement of Students

Table 6 lists the result of the mediating effect analysis by the bootstrap method, where *X* is the independent variable (MVPA) and Y is the dependent variable (mathematics achievement). The direct effect of MVPA on mathematics achievement in males and females was significant (95% confidence interval: 1.3906–3.4871 and 1.5650–5.4291, respectively). The indirect effect of each mediating variable was not significant; thus, MVPA has no mediating effect on mathematics achievement.

#### 3.4.2. Analysis of the Mediating Effect of Step on Mathematical Achievement

Table 7 presents the result of the mediating effect analysis by the bootstrap method, where *X* is the independent variable (step) and Y is the dependent variable (mathematics achievement). The direct effect of step on mathematics achievement of male and female students is significant (95% confidence interval: 0.0034–0.0059 and 0.0039–0.0065, respectively). However, the indirect effect of each mediating variable is not significant; thus, step has no mediating effect on mathematical achievement.

Analysis of the mediating effect of MVPA on mathematical achievements is listed in Table 6.

Analysis of the intermediary effect of step on mathematical achievement is listed in Table 7.

## 4. Discussion

We have used empirical research to explore the correlation between PA and AA and it has been found that PA could affect students' mathematics performance to a certain extent. In males, the multiple LR equation has shown that mathematics scores were related to MVPA steps and sustained attention. In females, the multiple LR equation has shown that mathematics scores were related to MVPA and steps. To summarize, PA has a direct effect on AA and there are no mediating effects between PA and three kinds of attention, namely, selective attention, sustained attention, and alternating attention.

One possible explanation of our findings is that individuals with more agile bodies have higher flexibility and achieve greater number of steps. Mathematics requires higher mental function and coordination between the hands and brain. The ability to solve mathematics problems requires using the brain and the using a paper, pen, and tools for calculations [21].

Moreover, MVPA has an effect on mathematics performance because proper PA could improve blood circulation, thereby fully mobilizing the functions of various organs of the body, including the brain [22]. PA has a positive effect on the central nervous system, such as enhanced flexibility of the central nervous system and coordination with various systems, improved ability to respond and keep the balance of the interaction between excitatory and inhibition processes, and improved regulation of the central nervous system and muscle activity of the body [23]. These effects could in turn make individuals more energetic, thereby improving learning efficiency [4]. Moreover, PA could help stabilize students' emotions, thus enabling students withstand greater academic pressure. A team of experts who studied the significance of PA in school-age children proved that active children and adolescents are more encouraged to improve their AA before, during, and after school because exercising and fitness contributed to the development of children's brain structure, function, and cognitive ability [15]. Moreover, middle school is the golden age of physical and intellectual development. For students, the burden of learning is not considered extremely heavy because they have much spare time. Moreover, the longer one engages in PA, the more exercise his or her body gets and their physical condition improves, which in turn provides sufficient energy and a strong body for learning. Exercise could also enrich students' extracurricular activities, stimulates the release of a variety of chemicals such as dopamine and endorphin, thus enhancing memory and intellectual development and promoting happiness. An optimistic attitude or a positive mental outlook is also conducive for improving learning ability.

BMI and aerobic fitness do not have any effect on mathematics scores because these obesity indicators have little effect on the students' willingness to exercise and exercise preferences, and the ability to solve mathematical problems is more related to the understanding and application of mathematical knowledge [16]. In this study, sustained attention levels did not show a mediating effect, and the analysis of sex differences showed contradicting results. Sustained attention could only be effective in predicting mathematical performance (direct effect and indirect effect) under certain conditions [21]. Moreover, sex differences in terms of persistent attention exist, which may explain the results of this study to some extent. Moreover, the absence of the mediating effect of sustained attention could be attributed to the fact that sustained attention is extremely limited. Even in the most ideal interference-free environment, an individual's attention span could only last for 20–22 min. The time required for measuring AA is much longer than the duration of the individual's attention; thus, the effect of sustained attention is extremely limited.

Therefore, the level of sustained attention did not produce a prominent effect in this study.

Furthermore, alternating attention was a form of selective switching attention. In our study, the effect of divergent attention on the student's learning process was not significant. For students, learning for or taking exams is what they usually have to face. The diversion of attention and demand for attention distribution are not high. The time for a person to complete a color test could only reflect his/her sensitivity to color. Thus, Stroop test results are not associated with mathematics scores. For selective attention, selective attention could be correlated with AA to a certain extent. However, AA could not be predicted with selective attention, which is consistent with the finding of our study. Moreover, although selective attention has no direct influence on AA, it has an indirect effect on working memory when it is needed, which is used as a mediating variable. Thus, AA could be influenced by the combination of sustained attention, alternating attention, and selective attention as mediating variables of PA. However, based on the BA, we found no mediating effects between PA and the three kinds of attention. Hence, whether selective attention, sustained attention, and alternating attention have direct or indirect effects on PA and AA requires further investigation, and future studies on other mediating variables between the three kinds of attention and AA are warranted. There are some limitations to this study. First, the sample in our study involved only one school. This study would need to be replicated in other areas in the USA and around the USA for greater transferability. Moreover, more factors regarding PA and psychology could be explored and considered for a more accurate statistical analysis.

## 5. Conclusions

This research study has carried out the empirical research on the identified population. The findings of the paper state that PA has a direct effect on AA. There are no mediating effects between PA and three kinds of attention, namely, selective attention, sustained attention, and alternating attention. No mediating effects are observed between PA and the three kinds of attention, namely, selective attention, sustained attention, and alternating attention. In males, the multiple LR equation has shown that mathematics scores were related to MVPA and sustained attention. In females, the multiple LR equation showed that mathematics scores were related to MVPA and steps. From the above findings, it can be concluded that the short time spent on physical activities during PE classes and sports related activities impacts positively on the academic performances of middle school students. It is found that there is a strong correlation between the physical activities and overall performance of the participant subjects. This is due to the hormones, which are released by the body during physical activities that relieve stress and increase concentration levels. This has resulted into the greater success in terms of mathematics scores of the middle school children. This study is applicable to all the age groups but in this research study, we are focusing on the school going kids and the impact of physical activities on the academic performance is remarkable as per the research study. The study of biomarkers also show that the relevance of physical activities on the overall performance of the students. The computational techniques like regression were used in predicting the performance on the basis of input activity data of the participants. The future study will concentrate on analyzing the impact of physical activities on the participants by considering the most relevant biomarkers of health analysis. We will introduce better computational techniques with different datasets to draw conclusive remarks to analyze the impact of physical activities on the academics. Furthermore, we will explore more factors related to PA and psychology in the future.

## Figures and Tables

**Table 1:** BMI classification of students in grades 7 and 8^∗^.

Variable	Boys (*n* = 93)	Girls (*n* = 83)	Total
Height (cm)	163.68 (7.506)	157.84 (6.865)	160.93 (7.761)
Weight (kg)	62.47 (17.145)	62.15 (15.928)	62.32 (16.536)
BMI	23.1591 (5.50100)	24.7992 (5.34419)	23.9326 (5.47407)

*Note*. Values are shown as mean (standard deviation).

**Table 2:** Sample characteristics for middle school students in grades 7 and 8.

school Section	Severe thinness		Thinness		Normal		Overweight		Obesity	
	Boys	Girls	Boys	Girls	Boys	Girls	Boys	Girls	Boys	Girls
7	≤13.8	≤13.6	<14.9	<14.9	14.9–21.7	14.9–22.7	>21.7	>22.7	≥25.8	≥27.2
8	≤14.3	≤14.0	<15.5	<15.4	16.4–22.6	15.5–22.6	>22.6	>23.5	≥26.9	≥28.2

**Table 3:** Distribution of BMI in male and female students in grades 7 and 8.

Gender		N	Percentage
Boys	Severe thinness	0	0
	Thinness	1	1.1
	Normal	46	49.5
	Overweight	24	25.8
	Obesity	22	23.7
	Aggregate	93	100.0
Girls	Severe thinness	0	0
	Thinness	2	2.4
	Normal	31	37.3
	Overweight	23	27.7
	Obesity	27	32.5
	Aggregate	83	100.0

**Table 4:** Regression coefficient of the final model of boys' regression equation.

Coefficient^a^					T	Significance
Model		Unnormalized coefficient		Normalized coefficient		
		B	Standard error	Beta		
Boys	Constant	5.839	5.855		0.997	0.321
	Step	0.004	0.001	0.535	6.293	0.0001
	MVPA	1.302	0.435	0.254	2.993	0.004
	Sustained attention	0.657	0.316	0.160	2.078	0.041

*Note*. ^a^Dependent variable: math.

**Table 5:** Regression coefficient of the final model of girls' regression equation.

Coefficient^a^					t	Significance
Model		Unnormalized coefficient		Normalized coefficient		
		B	Standard error	Beta		
Girls	Constant	6.327	4.504		1.405	0.164
	Step	0.005	0.001	0.594	7.649	0.0001
	MVPA	1.610	0.406	0.308	3.963	0.0001

*Note*. ^a^Dependent variable: math.

**Table 6:** Analysis of the mediating effect of MVPA on mathematical achievements.

Direct effect of *X* on *Y*			Indirect effect (s) of *X* on *Y*								
	Boys	Girls		Boys				Girls			
				Effect	BootSE	BootLLCI	BootULCI	Effect	BootSE	BootLLCI	BootULCI
Effect	2.4389	3.4970	Total	0.0069	0.2879	−0.5624	0.5947	−0.5922	0.7934	−2.3109	0.8381
se	.5273	0.9701	Aerobic fitness	0.0811	0.2346	−0.4045	0.5437	−0.4304	0.7334	−2.0494	0.8330
*t*	4.6252	3.6050	Selective attention 1	−0.0510	0.0985	−0.2617	0.1398	−0.0258	0.0747	−0.1861	0.1285
*p*	.0001	0.0006	Selective attention 2	0.0224	0.1208	−0.1810	0.3193	−0.1083	0.1589	−0.4627	0.1781
LLCI	1.3906	1.5650	Sustained attention	−0.0351	0.1058	−0.2668	0.1783	−0.0276	0.2042	−0.4211	0.4213
ULCI	3.4871	5.4291	Alternating attention	−0.0106	0.0746	−0.1884	0.1379	0.0001	0.1143	−0.2600	2378

**Table 7:** Analysis of the intermediary effect of step on mathematical achievement.

Direct effect of *X* on *Y*			Indirect effect (s) of *X* on *Y*								
	Boys	Girls		Boys				Girls			
				Effect	BootSE	BootLLCI	BootULCI	Effect	BootSE	BootLLCI	BootULCI
Effect	0.0046	0.0052	Total	0.0001	0.0003	−0.0006	0.0007	0.0005	0.0003	−0.0001	0.0011
se	0.0006	0.0007	Aerobic fitness	0.0001	0.0002	−0.0004	0.0005	0.0006	0.0003	0.0001	0.0011
*t*	7.2668	7.9861	Selective attention 1	0.0001	0.0001	−0.0001	0.0004	0.0001	0.0001	−0.0003	0.0001
*p*	0.0001	0.0001	Selective attention 2	0.0001	0.0002	−0.0004	0.0003	0.0001	0.0001	−0.0003	0.0002
LLCI	0.0034	0.0039	Sustained attention	−0.0001	0.0001	−0.0003	0.0001	0.0001	0.0001	−0.0002	0.0002
ULCI	0.0059	0.0065	Taril_M2	0.0001	0.0001	−0.0002	0.0002	−0.0001	0.0002	−0.0004	0.0003

## Data Availability

The data used to support the findings of the study can be obtained from the first author upon request.

## References

[1] Judge S., Jahns L. (2007). Association of overweight with academic performance and social and behavioral problems: an update from the early childhood longitudinal study. *Journal of School Health*.

[2] Kaur M., Kadam S. (2021). Bio-inspired workflow scheduling on HPC platforms. *Tehnički Glasnik*.

[3] Howie E. K., Pate R. R. (2012). Physical activity and academic achievement in children: a historical perspective. *Journal of Sport and Health Science*.

[4] Kaur M. Elitist multi-objective bacterial foraging evolutionary algorithm for multi-criteria based grid scheduling problem.

[5] Chaddock L., Pontifex M. B., Hillman C. H., Kramer A. F. (2011). A review of the relation of aerobic fitness and physical activity to brain structure and function in children. *Journal of the International Neuropsychological Society*.

[6] Zhang W., Kaur M. (2022). A novel QACS automatic extraction algorithm for extracting information in blockchain-based systems. *IETE Journal of Research*.

[7] Mule N. M., Patil D. D., Kaur M. (2021). A comprehensive survey on investigation techniques of exhaled breath (EB) for diagnosis of diseases in human body. *Informatics in Medicine Unlocked*.

[8] Kaur M., Sakhare S. R., Wanjale K., Akter F. (2022). Early stroke prediction methods for prevention of strokes. *Behavioural Neurology*.

[9] Kaur M., Jadhav A., Akter F. (2022). Resource selection from edge-cloud for IIoT and blockchain-based applications in industry 4.0/5.0. *Security and Communication Networks*.

[10] Boreham C., Riddoch C. (2001). The physical activity, fitness and health of children. *Journal of Sports Sciences*.

[11] Curtis G. L., Chughtai M., Khlopas A. (2017). Impact of physical activity in cardiovascular and musculoskeletal health: can motion be medicine?. *Journal of Clinical Medicine Research*.

[12] Khan N. A., Hillman C. H. (2014). The relation of childhood physical activity and aerobic fitness to brain function and cognition: a review. *Pediatric Exercise Science*.

[13] Tanha T., Wollmer P., Fedorowski A., Thorsson O., Karlsson M. K., Dencker M. (2016). Correlation between physical activity, aerobic fitness and body fat against autonomic function profile in children. *Clinical Autonomic Research*.

[14] Ryu J.-S., Chung H. R., Meador B. M., Seo Y., Kim K.-O. (2021). The associations between physical fitness, complex vs simple movement, and academic achievement in a cohort of fourth graders. *International Journal of Environmental Research and Public Health*.

[15] Tomporowski P. D., Davis C. L., Miller P. H., Naglieri J. A. (2008). Exercise and children’s intelligence, cognition, and academic achievement. *Educational Psychology Review*.

[16] Harvey S. P., Lambourne K., Greene J. L., Gibson C. A., Lee J., Donnelly J. E. (2018). The effects of physical activity on learning behaviors in elementary school children: a randomized controlled trial. *Contemporary School Psychology*.

[17] Diamond A., Ling D. S. (2016). Conclusions about interventions, programs, and approaches for improving executive functions that appear justified and those that, despite much hype, do not. *Developmental Cognitive Neuroscience*.

[18] Rasberry C. N., Lee S. M., Robin L. (2011). The association between school-based physical activity, including physical education, and academic performance: a systematic review of the literature. *Preventive Medicine*.

[19] Pindus D. M., Drollette E. S., Scudder M. R. (2016). Moderate-to-vigorous physical activity, indices of cognitive control, and academic achievement in preadolescents. *The Journal of Pediatrics*.

[20] Ishihara T., Morita N., Nakajima T., Okita K., Yamatsu K., Sagawa M. (2018). Direct and indirect relationships of physical fitness, weight status, and learning duration to academic performance in Japanese schoolchildren. *European Journal of Sport Science*.

[21] Barbosa A., Whiting S., Simmonds P., Scotini Moreno R., Mendes R., Breda J. (2020). Physical activity and academic achievement: an umbrella review. *International Journal of Environmental Research and Public Health*.

[22] Rodriguez C. C., Camargo E. M. D., Añez C. R. R., Reis R. S. (2020). Physical activity, physical fitness and academic achievement in adolescents: a systematic review. *Revista Brasileira de Medicina do Esporte*.

[23] Howie E. K., Pate R. R. (2012). Physical activity and academic achievement in children: a historical perspective. *Journal of Sport and Health Science*.

[24] Kayani S., Kiyani T., Wang J., Sánchez M. L. Z., Kayani S., Qurban H. (2018). Physical activity and academic performance: the mediating effect of self-esteem and depression. *Sustainability*.

